# Notes on the Voluntary Notification of Phthisis, and on the Municipal Attitude in Bristol in Regard to the Disease

**Published:** 1905-12

**Authors:** D. S. Davies

**Affiliations:** Medical Officer of Health, Bristol


### NOTES ON THE VOLUNTARY NOTIFICATION OF PHTHISIS, AND ON THE MUNICIPAL ATTITUDE IN BRISTOL IN REGARD TO THE DISEASE.

BY

D. S. DAVIES, M.D. Lond.,
*Medical Officer of Health, Bristol.*

IN January, 1899, I had the honour of introducing the annual discussion on the Etiology and Prevention of Tuberculosis before the Bath and Bristol Branch of the British Medical Association.[1] Owing to the recent formation of the National

[1] *Brit. M. J.*, 1899, i. 835.

Association for the Prevention of Consumption and the renewed public interest taken in the matter, the occasion was a favourable one for the initiation of fresh effort in the city.

In April of the same year I prepared for the Health Committee a special report on tuberculosis, with a consideration of local conditions and preventive measures. This now forms a useful landmark from which to estimate recent progress.

In this report I pointed out that, during the ten years 1889-98 inclusive, the seven principal zymotic diseases had caused 4,673 deaths in Bristol, while pulmonary consumption alone caused 3,520, or, adding in the deaths from other tubercular diseases, those due to tuberculosis generally amounted to a total of 4,876 in the same period, or more than all the "zymotic" diseases put together. The total deaths from all causes in the same period amounted to 43,141, so that about one death in every nine was due to some form of tubercle, and one in every twelve due to consumption of the lungs.

At the same time I summed up the position thus:—"It is definitely established that tuberculosis is primarily due to a specific organism (the tubercle bacillus), while weakness of constitution and unfavourable conditions of life, habitation, and environment play an important but secondary part in its causation and spread. Transmission of the pulmonary form of the disease occurs, as a rule, directly by means of the dried sputum, from person to person, and may be controlled by the observance of precautions in regard to the patient and the room he inhabits, and the securing of disinfection in regard to the premises. Certain forms of the disease appear also to be caused, especially in the young, by milk from cows similarly affected, and, in smaller degree, by meat from affected animals. Prevention thus lies partly in the hands of the local sanitary authority, partly in the hands of the medical attendant, and partly in the hands of the patient."

In January, 1899, the Council of the British Medical Association issued a report which stated their conclusions as to the conditions to be aimed at in municipal control in regard to the prevention of tuberculosis, and I dealt with these conclusions *seriatim*, showing how far the city of Bristol then complied with the conditions. It may be instructive to reconsider the conclusions and to estimate the progress that has been made.

1. DWELLINGS.

*Every house newly erected should be on a dry site and dry foundation (natural or artificial), and have sufficient space around it to allow of free access of air and sunlight. These conditions should be enforced by regulations of a national and not of a local (by-laws) character.*

The local by-laws were noted in 1899 as satisfactory, guarding against dampness and also providing for air circulation. The Public Health Acts Amendment Act 1890 is in force in Bristol, and prohibits building on noxious sites. The Model By-laws providing, *inter alia*, for the artificial elevation of low or damp sites, are not in force in Bristol.

2. MEAT.

*Properly qualified men should be appointed as meat inspectors.*

Two meat inspectors with good experience were on duty in 1899; no further appointment has yet been made to meet the recent extensions.

3. *Powers should be obtained from Parliament as follows:—*

(A) SLAUGHTER HOUSES. (In all County Boroughs.)

(1) *To afford facilities for providing public slaughter houses.*

(2) *To prohibit slaughter for gain except in a public slaughter house, after provision of such slaughter house.*

(3) *Elsewhere all licences to be renewable annually, and revocable easily.*

(4) *Compulsory examination of every cargo of imported meat, the charge to be borne by the importer.*

No public slaughter house had been provided in 1899. The question was fully reported on and the provision of one advised in 1895. No public slaughter house has been provided.

Licences granted after the adoption of the Public Health Acts Amendment Act 1900 are limited to twelve months, and may be revoked by a Court of Summary Jurisdiction upon conviction of an offence under the Act. This gives some control.

The importations into Bristol are practically entirely of live cattle. If dead meat were imported no opportunity of inspection would be afforded under present conditions before it reached the shops.

There are 116 private slaughter houses in the city, and two belonging to the Docks Committee. The number of inspectors is obviously insufficient to maintain efficient control over so many scattered slaughter houses—the area of the city, which in 1897 was 4,661 acres, has now increased (1905) to 17,289 acres.

(B) MILK.

(1) *The minimum air space in cowsheds should be fixed at 800 cubic feet per cow.*

(2) *Power should be given to local authorities to inspect systematically or cause to be inspected all dairies and cowsheds from which milk is sent into their districts, and to exclude the milk if the application of the tuberculine test to any cow suspected of being tuberculous be refused, such inspection to be carried out by the Medical Officer of Health, with the assistance of a veterinary officer.*

(3) *Power to take samples of milk from any particular cow or cows.*

(4) *Power to prohibit the entrance of milk into any district if there are reasonable grounds for suspecting it to be tuberculous.*

(5) *All tumours (or affections) of the udders should be notifiable. If any cow is ordered to be slaughtered, there should be power to pay compensation if the cow proves not to be tuberculous.*

(6) *Imported milk (and dairy products) should be examined at the port of entry, the expense to be borne by the importer.*

When I reported in 1899 upon these suggestions, I pointed out that, while the regulations in force in the city prescribed due cubic space for cowsheds and powers of inspection and control for dairies situate within the city, they gave no powers available in regard to dairies, etc., situated outside the city, and no powers authorising any special action with regard to tuberculosis in cattle. The only powers then granted in regard to outside dairies were under the Infectious Disease Prevention Act 1890, section 4, in the case of milk causing "infectious disease."

The Bristol Corporation Act 1905, which received the Royal Assent on August 11th, contains, however, several clauses, introduced by the Health Committee, as to milk supply and food, which show a distinct advance in our preventive measures.

The following are, briefly, the new provisions:—

PART VI.

*Clause 41.* Penalty of ten pounds on any person for knowingly selling within the city milk of any cow suffering from tuberculosis of the udder.

*Clause 42.* Penalty of five pounds on any person neglecting to separate a cow suffering from tuberculosis of the udder from the rest of the herd.

*Clause 43.* Penalty of forty shillings on every dairyman supplying milk within the city for neglect to send notice to the Medical Officer of Health that he has a cow suffering from tuberculosis, or suspected of so suffering.

*Clause 44.* Power given to Medical Officer of Health to take within the city samples of milk, also to take samples outside the city after obtaining a Justice's Order.

*Clause 45.* Power given to Medical Officer of Health to inspect, with the aid of a veterinary surgeon, cows in any dairy within the city for tuberculosis of udder, and to secure immediate samples of milk; also power to deal with a dairy within or without the city suspected of causing, or of being likely to cause, tuberculosis to persons residing within the city; with an accompanying penalty of five pounds for obstruction.

Acting on a recommendation in my report, the Health Committee authorised a systematic examination of the milk from all farms supplying the city, and this was carried out by Professor Délépine, of Manchester. The samples were taken from the milk as it was brought into the city for delivery. The results were more favourable than had been anticipated, for out of seventy-four samples examined by the inoculation test only four showed the presence of tuberculosis.

4. Pathological Diagnosis and Disinfection.

(1) *All local authorities should make arrangement for the bacterial diagnosis of tuberculosis at the expense of such authorities.*

This had not been done in 1899, but arrangements are now (1905) in working order by which the bacterial diagnosis of tuberculosis is made at the cost of the Corporation for medical practitioners in regard to cases of Consumption occurring within the city.

(2) *Local authorities should undertake, free of charge, the disinfection of houses in which a tuberculous patient has resided, together with the bedding, clothing, and other articles capable of retaining the infection.*

This has been offered in the annual reports from 1891 onwards, but very little advantage was taken of it until the recrudescence of public interest in tuberculosis. During the year 1905, up to November 14th, 315 houses have been dealt with in this way by the inspectors.

Notification.

In September, 1903, the City Council approved of the initiation of a voluntary system of notification of phthisis. This came into force on September 7th, 1905, and 235 notifications had been received up to November 1st.

5. Treatment.

*Poor Law Guardians and Local Sanitary Authorities should be encouraged to provide suitable accommodation for the treatment of consumptive patients.*

In 1899 I had to report that no provision had been made locally, and expressed the opinion that "much good may no doubt result from properly directed treatment in the early stages of the disease."

I can now record (1905) that the Bristol City Council, on the recommendation of the Health Committee, have made the following arrangements for dealing with consumption in the city:—

WINSLEY SANATORIUM.

They have contributed a sum of £5,000 towards the acquirement of twenty beds in the Sanatorium, and they contribute a further sum of £1,300 annually towards the maintenance of these beds, which are reserved at their disposal. The Winsley Sanatorium was opened in December, 1904. The first Bristol case was admitted on February 27th, 1905. Fifty-three cases have been admitted up to November 1st, out of 147 applications received. Thirty-four cases have been discharged, in nineteen of these considerable improvement, and in nine others some degree of improvement, was recorded. In the remaining six no improvement was reported on discharge. When the early cases were admitted the applications were comparatively few, so that some of the beds became filled with less suitable cases than happens now that the applications are considerably in excess of the available beds. It is too early in our experience to criticise the results obtained up to date, and before venturing to do so the report of the medical officer to the institution, which will be issued for the year, must be carefully studied.

There is in my mind no doubt whatever that sanatorium treatment is *one* of the essential factors in the municipal dealing with consumption, a factor without which all other available measures would fail in great part to exert much useful good.

The good effect of sanatorium treatment consists partly in the result attained with the actual patient in building up and attuning his inherent resistance, so that in favourable cases the continued growth of the bacillus may be inhibited, and he may proceed to recovery, which pathological examination has demonstrated not to be an uncommon occurrence in tuberculosis; but far more wide-reaching is the effect of the training which an intelligent patient retains, not only for the future conduct of his own life, but by precept and example for the lives of others.

In tuberculosis, as in other parasitic micro-organismal diseases, no one factor can be assigned as the "cause"—neither "housing," nor "habits," nor "methods of work," nor "feeding," nor "hereditary" tendency, nor "dampness of site and soil"—but each may be contributory, and each in turn must be dealt with to ensure success.

The present arrangements of the Guardians for dealing with consumptive cases are as follows:—

*In the Stapleton Workhouse.*—A large ward containing thirty-six beds is reserved for male consumptives, special alterations having been made to the windows with a view to the treatment of this class of case.

*In the Southmead Workhouse.*—The Guardians are now utilising the temporary hospital attached to the Southmead Workhouse, which was recently acquired on the dissolution of the Barton Regis Union, for the treatment of female consumptives and children. This temporary building contains twenty-two beds.

BYE-LAW AGAINST SPITTING.

In 1903 the Bristol City Council adopted the following Bye-law:—"A person shall not spit on the floor, side or wall of any public carriage, or of any public hall, public waiting-room, or place of public entertainment, whether admission thereto is obtained upon payment or not. Any person offending against this Bye-law shall be liable to a fine not exceeding £1."

Copies of this Bye-law and other notices have also been widely distributed to publicans, owners and occupiers of workshops and workplaces, and in various public places.

All recent contracts for the supply of meat and milk to the Bristol City Hospitals have been framed upon lines protective against tuberculosis.

*The Local Sanitary Authorities should also take steps to diminish overcrowding in tenement houses and cottages.*

The local regulations provide as follows:—

BYE-LAWS.

| | Cubic feet per head. |
|---|---|
| Common lodging houses— | |
| Sleeping rooms (2 children under 10 = 1 lodger) | 300 |
| Houses let in lodgings— | |
| Rooms used exclusively as a sleeping room ... | 300 |
| Room used as a sleeping apartment, but not exclusively for that purpose ... ... ... | 400 |
| Workshops. Factory and Workshop Act 1901— | |
| For each employé ... ... ... ... ... ... ... ... | 250 |
| Ditto, during overtime... ... ... ... ... ... ... | 400 |

THE OUTLOOK.

In view of the recently-awakened public and municipal interest in consumption, the outlcok is, I think, distinctly favourable. Although this disease still holds an unfortunate pre-eminence amongst fatal micro-organismal diseases, it is satisfactory to note that since 1851 there has been a consistent and continuous reduction in the phthisis mortality in England and Wales, as shown here:—

| | | | | | |
|---|---|---|---|---|---|
| 1851 - 60 | = | 2.7 | 1881 - 85 | = | 1.8 |
| 1861 - 70 | = | 2.5 | 1886 - 90 | = | 1.6 |
| 1871 - 80 | = | 2.1 | 1891 - 95 | = | 1.5 |

There is good reason to hope that the diminution may be not only maintained but improved upon.

GENERAL MEASURES.

*Medical Officers of Health are advised to draw up leaflets for distribution, describing the precauticns to be taken to prevent tuberculosis, and also the precautions to be taken with patients to prevent the spread of infection, etc.*

Such instructions were first issued in Bristol in 1891, reprinted in 1892, 1893 and 1894, and were reissued in 1899.

In September, 1903-4, by direction of the Council, 80,000 copies of a revised pamphlet, which has appeared in the *Bristol Medico-Chirurgical Journal*, were issued through every district in the city, and as far as possible to every house; this was done on two occasions, with an interval of three months between. The distribution of these leaflets was first undertaken by the members of the St. John's Ambulance Brigade (City of Bristol Corps), who also undertook to provide Dettweiler Sputum Flasks, free of cost, to consumptive patients unable to afford the purchase. The Health Committee supply gratuitously, to patients recommended by the Corps, the necessary disinfecting solution for use in these flasks. The Health Committee have also in contemplation the provision of sputum flasks for certain deserving cases not reached by the St. John's Ambulance Association.

